# Inference of a plume conduit beneath the Réunion Island from 3D migration of Ps conversions from the mantle transition zone

**DOI:** 10.1038/s41598-025-94831-3

**Published:** 2025-03-29

**Authors:** B. Padma Rao, M. Ravi Kumar

**Affiliations:** 1 MoES-National Centre for Earth Science Studies, Govt. of India, Thiruvananthapuram, India; 2 CSIR-National Geophysical Research Institute, Hyderabad, India

**Keywords:** Seismology, Geodynamics

## Abstract

The Réunion hotspot is the best example of a primary plume, manifested as intraplate-volcanism, a large igneous province and a geochemical anomaly. In this study, we investigate the mantle transition zone (MTZ) structure beneath the Réunion Island using 3D-migration of P-Receiver functions, to decipher the effect of the plume on the MTZ and its architecture. Results indicate a thin MTZ in the regions surrounding the Réunion, like Madagascar and its vicinity, eastern and south-eastern sides of the Réunion, suggesting high-temperature anomalies within, caused by the plume. Interestingly, we detect a depressed 410 km discontinuity exactly beneath the Réunion hotspot and a broader depression of 660 km discontinuity within and regions in its proximity. These maiden results shed-light on the high-temperature anomalies in the mid-mantle, probably sourced from the Réunion plume and provide evidence for the Majorite-garnet phase transformation at 660 km discontinuity. We postulate that an ascending Réunion plume has initially hit the 660 km discontinuity, got horizontally spread and further progressed to the 410 km discontinuity as a columnar structure.

## Introduction

Hotspots, basaltic in composition, are the surface expressions of plumes that emanate from the deeper mantle as columns of hot rock. Although the source depth of plumes is debated, it has been speculated that the 200 to 300 km thick $\hbox {D}^{\prime \prime }$ layer just above the core-mantle boundary (CMB) may aid in the formation of hot plumes, due to transfer of heat from the core. Plumes are one of the major causative factors for continental breakup, massive flood basalt provinces, intraplate volcanism, and a chain of volcanic islands^1^. The Réunion hotspot is the best example of a primary plume^2–7^ that impinged the lithosphere and produced the Deccan flood basalts of India at $\sim$65 Ma. The plume is currently traced beneath the Réunion Island to the east of Madagascar in the Indian Ocean. The active Piton de la Fournaise volcano could be the surface expression of a long-lasting mantle plume and the southernmost point of this intraplate hotspot track.

Several studies were conducted to decipher the architecture, surface impact and geodynamical implications of this plume, especially using data from the RHUM-RUM project, under which 57 ocean bottom seismometers and 23 temporary land stations were installed. Using waveforms from these stations, Mazzullo et al.^8^ generated a three-dimensional anisotropic shear wave velocity model with a depth resolution down to 300 km. Their results capture signatures of the Réunion hotspot in terms of a slow velocity anomaly starting at 50 km below the Rodrigues ridge, in the depth range of 150–300 km beneath the Mascarene Basin. Also, they proposed a clear connection between plume upwelling and the Central Indian Ridge, based on the east-west orientation of anisotropy^8–10^. Further, a regional tomography model of the Indian Ocean, constrained to a depth of 1200 km^11,^ suggests that the rising plume has a broad head in the upper mantle and a narrow tail at deeper depths, which is likely fed by the African Large Low Shear Velocity Province (LLSVP). A recent study by Tsekmistrenko et al.^12^ presented a comprehensive whole mantle tomographic model derived from joint inversion of three types of P-wave observations. The first dataset, derived from the ISC-Engdahl, van der Hilst, and Buland (EHB) catalog, provides a conventional global P-wave model that primarily illuminates the mid-mantle. The second dataset, which is methodologically new, involves core-diffracted P waves (Pdiff) and offers constraints on the lowermost mantle on a global scale. The third dataset consists of 55,657 teleseismic multi-frequency travel times, measured using the dense RHUM-RUM array. Models derived from this dataset specifically resolve the upper half of the mantle beneath the oceanic Réunion hotspot. Their findings reveal the generation of three branches that extend outward and upward towards different Indo-Australia hotspots from the central, dense trunk beneath  1500 km depth. These branches correspond to vertically rising plumes or proto-plumes that got detached in a staggered, linear sequence from a low-velocity zone at the core-mantle boundary. As a plume reaches the viscosity transition between the lower and upper mantle, it generates a classic plume-head/plume-tail structure. Plenty of other studies have been carried out over the last few years to thoroughly investigate the seismic structure of the upper mantle and anisotropy beneath and around the hotspot, using various techniques like surface wave tomography^8^, ambient noise tomography^13^ and teleseismic shear-wave splitting^9^. In addition, the lower mantle structure has also been investigated through body wave tomography^14^. However, the effect and/or degree of impact of the rising plume on the mantle transition zone (MTZ) is less studied, especially beneath the Réunion hotspot.

The mantle transition zone (MTZ) marked by phase changes of Olivine at 410 km and 660 km discontinuities, is sensitive to the thermal state of the mantle. The depths to these interfaces vary depending on many factors, especially the temperature and composition of rocks underneath the region. In general, a thick MTZ with shallowing and deepening of 410 km and 660 km discontinuities respectively, is observed in cold environments such as those containing slabs, due to the opposite nature of the Clapeyron slopes of these two discontinuities^15^. Moreover, a thin MTZ is observed with deepening and shallowing of 410 km and 660 km discontinuities in hot conditions such as those generated by plumes. The temperature and pressure conditions around 660 km depth support non-Olivine i.e., Majorite-garnet phase transformations at the 660 km discontinuity. This discontinuity deepens in the presence of hot material due to a positive Clapeyron slope of the garnet phase transformation^16–18^. Therefore, clear imaging of these discontinuities enables deciphering the effect of a plume on the MTZ and its structure. In the present study, we used P-receiver functions and 3D-migration techniques to investigate the MTZ beneath the Réunion hotspot and the surrounding regions.

## Data and method

Waveform data from 113 broadband seismic stations are collated in such a way that the piercing points of the Ps conversions from the MTZ discontinuities locate at and around the Réunion Island. Out of the 113 stations, 60 are operated under the RHUM-RUM (Réunion Hotspot and Upper Mantle-Réunions Unterer Mantel, code YV) experiment^19,20^, which consists of 40 Ocean bottom seismometers (OBS) and 20 land stations. The RHUM-RUM experiment had deployments of OBS from the German DEPAS (Deutscher Geräte-Pool für Amphibische Seismologie) and French INSU (Institut national des sciences de l’Univers) pools, between October 2012 and December 2013. This experiment is designed to detect the presence or absence of plumes beneath the Réunion Island^8–10,19^. In this study, we utilized the orientations of OBS from the results published by Trabattoni et al.^21^ and Scholz et al.^22^. Scholz et al.^22^ determined the OBS orientations using teleseismic events, while Trabattoni et al.^21^ inferred the orientations with an accuracy better than 1°, through analysis of ship noise. Where data from Trabattoni et al.^21^ were unavailable, we adopted the orientations calculated by Scholz et al.^22^ to orient the OBS data. The remaining 53 stations, were operated under various networks viz., PF^23^, YA^24^, ZF^25^ and 3E^26^. In the present study, only those waveforms of earthquakes in the epicentral distance range of 35° to 95° with magnitude $\ge$ 5, listed in the CMT Catalog^27,28^, are considered. Further, the Signal to Noise Ratio (SNR) is calculated by division of the mean envelope amplitude of the signal on the vertical component within a time window of 30 s after the theoretical arrival time of the P wave and noise within a time window of 30 s on the same component before the arrival. Later, the waveforms having SNR $\ge$ 2 are only considered for analysis. These criteria resulted in 20,453 waveforms from 5420 earthquakes, recorded at 106 broadband seismological stations.

**Fig. 1:**
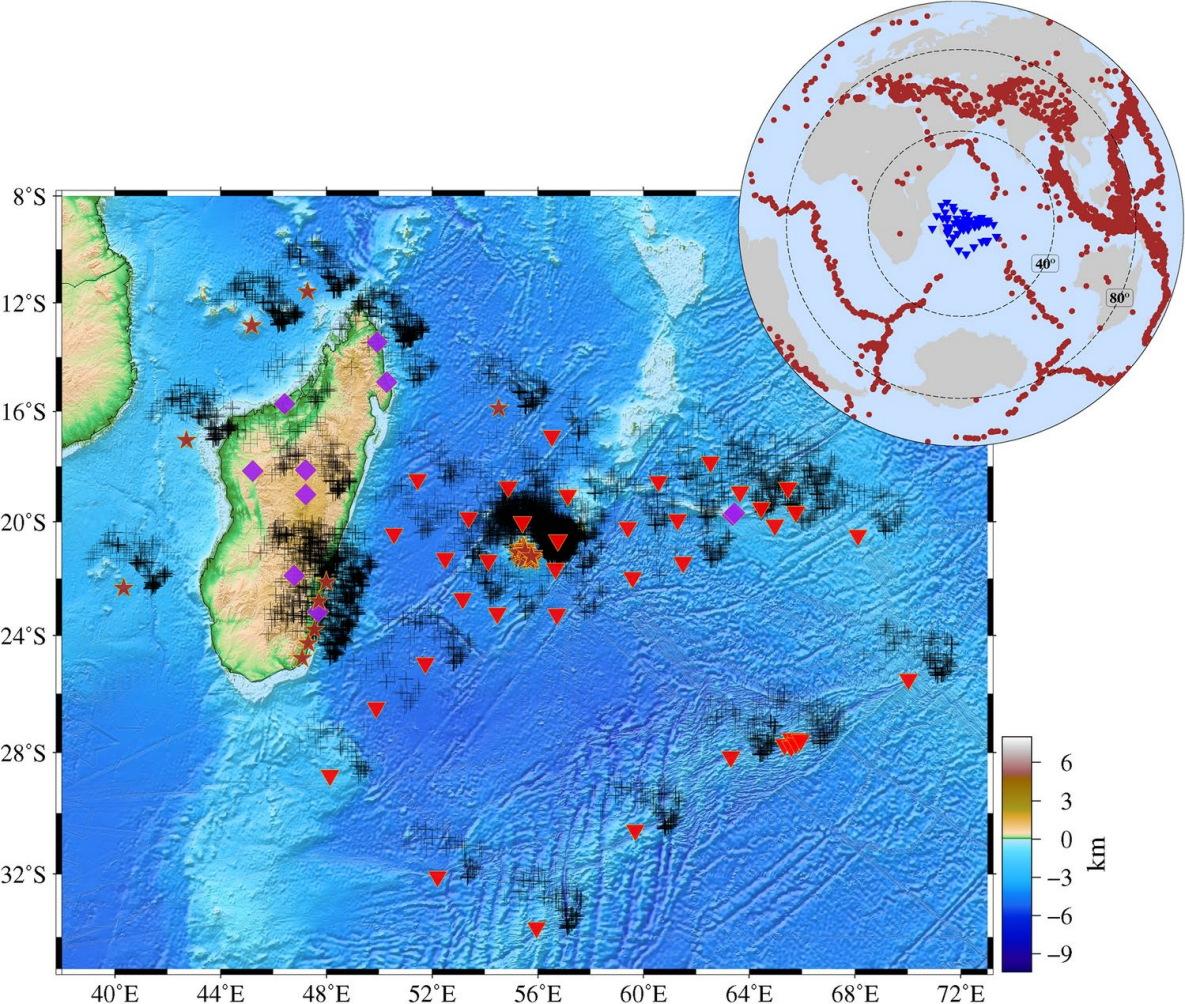
Locations of Ocean bottom seismometers (red inverted triangles), land seismometers (brown stars), land seismometers whose data is used by Rao et al.^31^ (purple diamonds) and piercing points of Ps conversions at 535 km depth (black crosses). Background indicates topography/bathymetry. Inset: Distribution of stations (blue inverted triangles) and earthquakes (brown circles) in the epicentral distance range of 35° to 95°, whose waveforms are utilized in the present study.

We adopted the extended-time multi-taper frequency domain cross-correlation (ET-MTRF) technique^29^ to compute the P-Receiver Functions (PRFs). In this method, we utilized the parameters including a time-bandwidth product of 2.5, three lowest-eigenvalue tapers, a window length of 10 s and a window overlap fraction of 75%, which creates a flat response. This method applies a series of short, overlapping tapers to window the time series across its length and sums the individual Fourier-transformed signals to produce a receiver function estimate, preserving the phase information for each sub-window. In the next stage, the computed PRFs are bandpass filtered between 0.05 and 0.5 Hz and distance moveout corrected using the IASP91 standard model with a reference epicentral distance of 67°, corresponding to a reference slowness of 6.4 s/°. This enables enhancement and identification of the low-frequency Ps conversions from the upper mantle transition zone discontinuities. Subsequently, the individual PRFs were visually checked to select good quality PRFs based on the following criteria: (i) distinguishable converted phases from the 410 km and 660 km discontinuities, (ii) no energy present before the zero-delay time, since prominent energy prior to the zero-delay time indicates improper deconvolution and (iii) absence of reverberatory phases. Accordingly, the PRFs that did not meet these criteria were discarded, resulting in the removal of 7110 PRFs from land stations and 1256 PRFs from OBS stations. This visual quality checking analysis resulted in selection of 12,087 good quality PRFs from 2828 earthquakes, whose waveforms were recorded at 72 stations (Fig. 1). Additionally, we calculated the differential slowness stacks^30^ of PRFs at individual stations to check the correctness of the identified P410s and P660s phases. This technique clearly distinguishes the converted phases from multiples, since the differential slowness is negative for converted phases and positive for the multiply reflected phases. Figure S1 shows example PRFs and the corresponding differential slowness stacks for two stations viz., MAYO and RR22. In addition, the processed data of 11 stations from Rao et al.^31^, which sample the study region, were also utilized in the present study (Fig. 1). These stations are operated under various networks, namely GE^32^, II^33^, XV^34^ and G^35^.

**Fig. 2:**
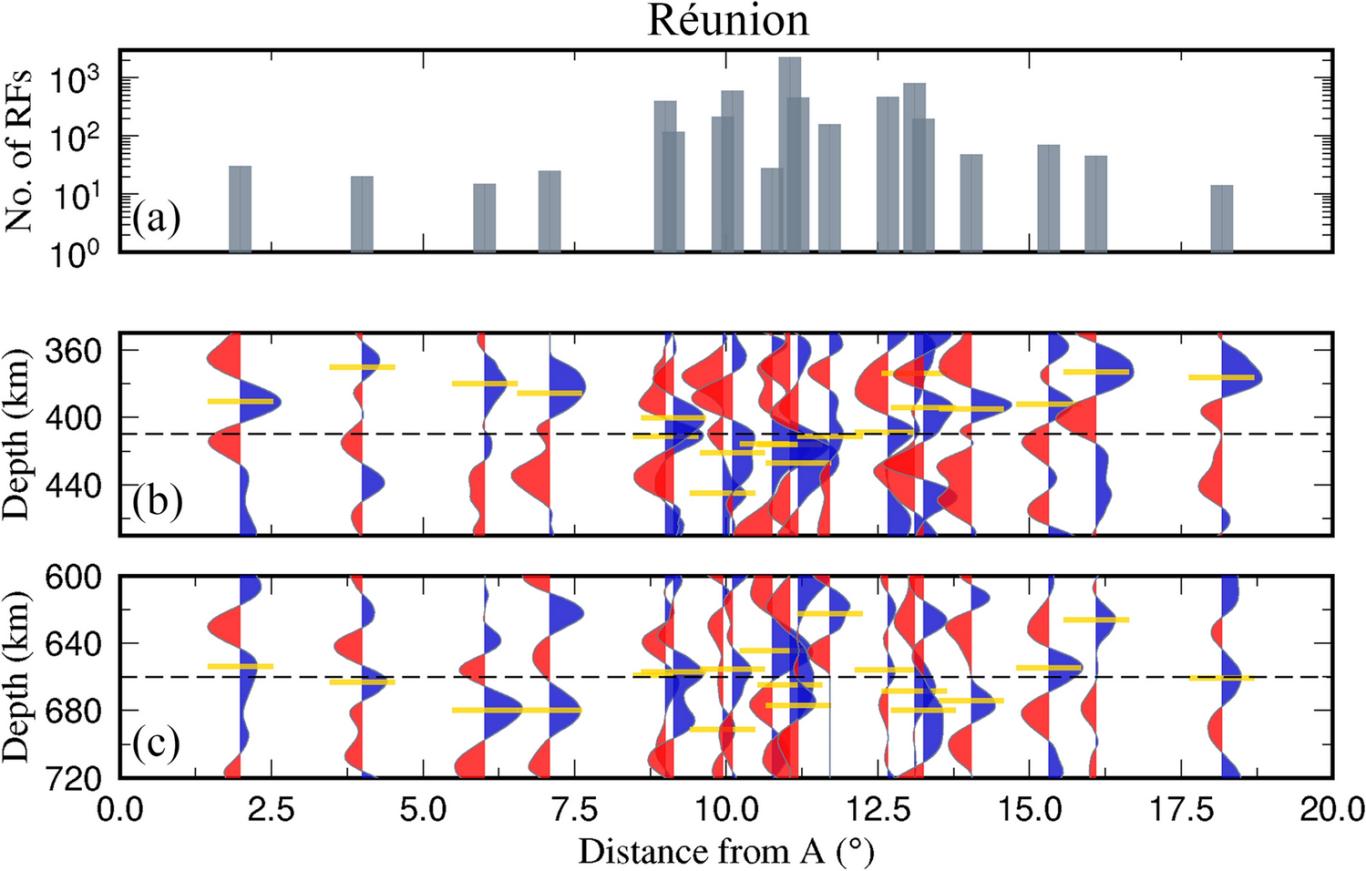
(**b**,**c**) PRFs, 3D-depth migrated and stacked using a 3D velocity model (GyPSuM), are plotted along profile AA*, marked in Fig. 3. Yellow horizontal lines in the (**b**) and (**c**) panels indicate depths to the 410 km and 660 km discontinuities respectively. (**a**) Shows the number of PRFs stacked in the corresponding grid.

The energy from different sources having different back-azimuths converges at a single point i.e., at a station, which makes it challenging to pick the weaker conversions from the upper mantle discontinuities on the moveout-corrected PRF stacks at individual stations. The move-out corrected PRF stacks at stations reflect the average structure within a $\sim$2° radius of the station, since the piercing points of Ps conversions at MTZ discontinuities are in this range. In order to minimize such complexities, enhance the quality and image the lateral variations of MTZ discontinuities, we divided the study region into circular grids of 1° radius with an overlap of 25% between consecutive grids. Since every grid may not have any piercing points or have only a very few, we have considered only those grids with more than 10 piercing points at the depth of 535 km, to obtain the stacked PRF representative of a grid. The stacking process enhances the conversions from the MTZ discontinuities since this sums the signals which are in-phase and suppresses those that are out-of-phase. To image the depths to the 410 km and 660 km discontinuities, it is essential to migrate the PRF time series to depth. The classical method for estimating the depths to the 410 km and 660 km discontinuities from the observed times uses a 1D velocity model. However, the 1D velocity model may not incorporate the high/low-velocity anomalies along the Fresnel zone of the P-S ray path, which affects the correct determination of depths to the MTZ discontinuities. Thus, we adopted a 3D-migration scheme utilizing the 3D-velocity model of P and S wave velocities, extracted from the GyPSuM^36^ global tomography model. Further, we also used the LLNL-G3D-JPS tomography model^37^ to compare the results. Later, the bootstrap resampling technique^38^ has been implemented to pick the depths to the 410 km and 660 km discontinuities and estimate their uncertainties in each grid. The bootstrap resampling is performed in such a way that the total number of PRFs in a grid are selected randomly, migrated to depth by computing the 3D ray geometry using the selected 3D tomography model and stacked to pick the depths to the 410 km and 660 km discontinuities automatically, based on the corresponding peak amplitudes. This process is repeated 200 times for each grid, with random numbers of different sequences and the mean and standard deviation of depths to the MTZ discontinuities are calculated. This process is applied for all the grids which sample the study region, to image the lateral variations. Later, the depth series PRFs from each grid were thoroughly checked to ensure that the depth migrated PRFs had clear converted phases from the 410 km and 660 km discontinuities and were noise-free after satisfying the piercing point criteria, to select the good quality ones. In some of the grids, we observed poor data quality though the grids contain a sufficient number of piercing points, probably due to oceanic noise. Most of the data in the present study are from OBS networks, in which the noise content is higher than that at the land stations, as expected. Therefore, the grids having good/fair quality Ps conversions from 410 km and 660 km discontinuities are only considered. Further, the 3D depth migrated PRFs corresponding to the grids from Rao et al.^31^, which sample the study region, were included for a better spatial sampling of the MTZ discontinuities. In total, we observed good quality 3D migrated PRFs in 66 grids. Examples of 3D-migrated PRFs of 4 grids and the corresponding mean and standard deviation of the depths to the 410 km and 660 km discontinuities are shown in Fig. S2. The 3D-depth migrated PRFs at all the grids sampling (i) the region beneath Madagascar and its surroundings (Reg-1), (ii) the region beneath the Réunion, Mauritius Islands and their surroundings (Reg-2) (Fig. 2), (iii) the eastern side of the Réunion and Mauritius Islands, sampling the oceanic region (Reg-3) and (iv) the south-eastern side of the Réunion Island, sampling the oceanic region (Reg-4), are shown in Figs. S3, S4, S5 and S6 respectively.

## Results and discussion

**Fig. 3:**
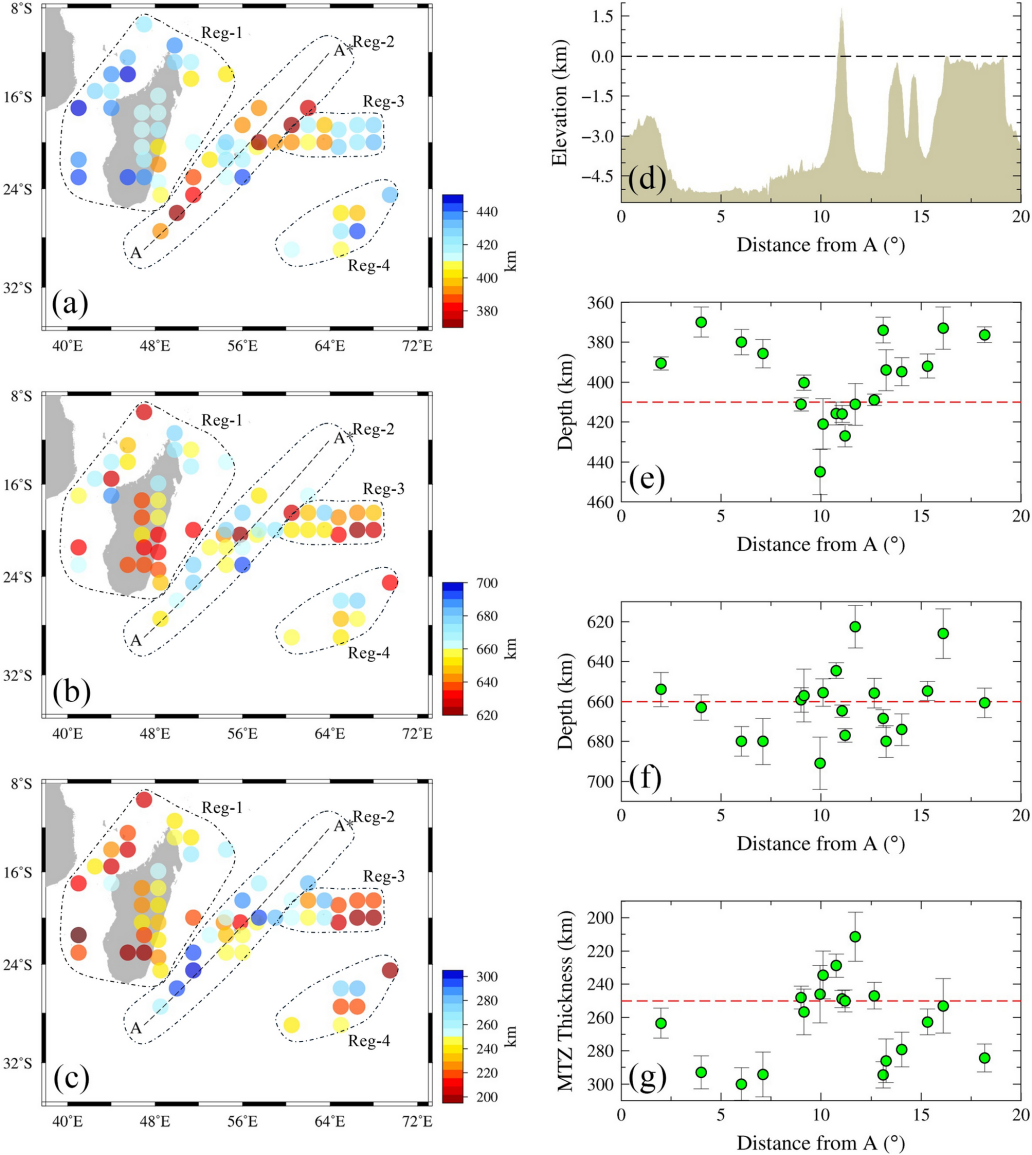
Variations in the depths to the (**a**) 410 km, (**b**) 660 km discontinuities and (**c**) mantle transition zone thickness, determined from stacking of PRFs depth migrated using the GyPSuM 3D velocity model. (**d**) Indicates the topography/bathymetry variations along the profile and the depths to the (**e**) 410 km discontinuity, (**f**) 660 km discontinuity and (**g**) mantle transition zone thickness. The dashed horizontal red line indicates the (**e**) 410 km depth, (**f**) 660 km depth and (**g**) global average of mantle transition zone thickness.

Distinguishable Ps conversions from the 410 km and 660 km discontinuities are observed for 66 grids. The stacks of PRFs 3D-migrated using the GyPSuM model indicate that the depth to the 410 km discontinuity varies from 370 km to 448 km and that to the 660 km discontinuity from 620 km to 691 km (Figs. S3, S8 and Table S1), with the transition zone thickness varying from 191 km to 300 km. These results indicate a depressed 410 km discontinuity and an elevated 660 km discontinuity beneath Madagascar and its surrounding region (Reg-1 in Fig. 3). Similar results are observed beneath the eastern (Reg-3 in Fig. 3) and south-eastern (Reg-4 in Fig. 3) sides of the Réunion and Mauritius Islands, sampling the oceanic part (Figs. 3 and Table S1). The MTZ is thin beneath these regions. The region beneath the Réunion, Mauritius Islands and their surroundings (Reg-2 in Fig. 3) shows average depths to the 410 km discontinuity varying from 370 km to 445 km and the depths to the 660 km discontinuity varying from 623 km to 691 km, with the MTZ thickness varying from 211 km to 300 km (Fig. 3 and Table S1). Further, the results are compared with the depths and the corresponding MTZ thicknesses determined from depth migrated PRFs, using another 3D velocity model i.e., the LLNL-G3D-JPS model. Similar results are obtained using the LLNL-G3D-JPS model (Fig. S7 and Table S2). The error in the measurements calculated with the help of bootstrap resampling technique^38^, reveals a value of $\sim$8 km, when the depth migration is performed using the GyPSuM global model. The observed error is almost similar when the depth migration is done using the LLNL-G3D-JPS model. This error may be due to the stacking of not so coherent conversions from the MTZ discontinuities, uncertainties in depth picking and the tomography model used for migration.

We present results along profile AA* crossing the Réunion hotspot and Mauritius regions (Reg-2 in Fig. 3), for a better understanding of variations in the depths to the 410 km and 660 km discontinuities and variation in the thickness of MTZ (Figs. 2 and 3). The depths to the 410 km discontinuity on the southwest and northeastern sides of the Réunion Island vary from 370 km to 411 km along profile AA*, indicating an elevated 410 km discontinuity. In contrast, the depths to the 410 km discontinuity beneath the region of Réunion vary from 416 km to 445 km, indicating a depressed 410 km discontinuity. Moreover, the majority of the depths to the 660 km discontinuity vary from 655 km to 691 km almost all along the profile except at a few endpoints, indicating a broadly depressed 660 km discontinuity. Interestingly, the results indicate depressed 410 and 660 km discontinuities exactly beneath the Réunion region. The majority of the MTZ thickness values along the profile vary from 253 km to 300 km on the southwestern and northeastern sides of the Réunion, indicating a thick MTZ. In contrast, the MTZ values beneath the Réunion vary from 229 km to 250 km, suggesting a slightly thinner MTZ where positive topography is seen, exactly beneath the Réunion. The trend of the thickness values corroborates with the results observed from the S410S-S660S differential travel times on a global scale^39^.

In general, the depths to the MTZ discontinuities are linked to phase transformations in the Olivine system. The topography and sharpness of these discontinuities are majorly sensitive to the compositional and thermal heterogeneities in the MTZ. Olivine (Ol) gets transformed into Wadsleyite (Wd) at the 410 km discontinuity, Wadsleyite (Wd) to Ringwoodite (Rw) at the 520 km discontinuity and then Ringwoodite (Rw) to Perovskite (Pv) + Magnesiowüstite (Mw) at the 660 km discontinuity. The Ol-Wd transformation at the 410 km discontinuity has a positive Clapeyron slope, while the Rw-Pv transformation at the 660 km discontinuity has a negative one^40–42^. This opposite nature of Clapeyron slopes of MTZ discontinuities suggests a thin MTZ in regions of hot anomalies and a thick one in regions having cold anomalies^43^. Moreover, studies carried out on the phase transformation of Majorite-garnet (Mj) to Perovskite (Pv) at 660 km depth^17^, show a positive Clapeyron slope. The temperature and pressure conditions around this depth support non-olivine i.e., Mj-Pv phase transformations at the 660 km discontinuity, which causes it to deepen under the influence of hot material, due to the positive Clapeyron slope of Mj phase transformation^16–18^. This indicates that this discontinuity deepens in hot regions and elevates in cold regions, similar to the behaviour of the 410 km discontinuity. Additionally, it has been proposed based on mineral physical studies that the non-olivine phase transformation at 660 km discontinuity plays a crucial role in understanding the compositional and thermal state of the MTZ in addition to the olivine phase transformation. A study by Hirose^17^ indicated that this phase transformation takes place in the pressure range of 1 to 1.5 GPa with 0.0013 GPa/°C (positive Clapeyron slope) and is broader than that of the olivine phase transformation. In order to understand the seismic nature of this transformation, plenty of studies have been undertaken^44–47^. Based on these results, it is concluded that this kind of phase transformation produces multiple, broad gradients and complex seismic phases.

The results sampling Madagascar and its surrounding region (Reg-1 in Fig. 3), the eastern (Reg-3 in Fig. 3) and south-eastern (Reg-4 in Fig. 3) side of the Réunion sampling the oceanic region, indicate a thin MTZ, majorly owing to a depression of the 410 km discontinuity and an elevation of the 660 km discontinuity. These kinds of topography changes could be due to the classical phase transformations i.e., Ol-Wd at the 410 km discontinuity and Rw-Pv phase transformation at the 660 km discontinuity. These results indicate signatures of a hot anomaly at MTZ depths. These hot anomalies are also noticeable in the global tomographic models as low shear wave velocity anomalies. Moreover, results along profile AA*, which crosses the Réunion Island indicate a slightly thin MTZ beneath the Réunion region owing to depressed 410 km, 660 km discontinuities and a slightly elevated 660 km discontinuity at a few grids beneath the region surrounding the Réunion. However, to the southwest and northeastern sides of the Réunion, a thick MTZ is seen along the profile, due to an elevated 410 km and a depressed 660 km discontinuity. These kinds of observations i.e., depression in both the MTZ discontinuities indicate a non-olivine phase transformation i.e., Mj-Pv phase transformation beneath the Réunion Island due to the presence of a hot anomaly, and a positive Clapeyron slope of Mj-Pv. The results also indicate an elevated 660 km discontinuity in a few grids surrounding Réunion Island, which could be due to an olivine phase transformation in addition to a non-olivine phase transformation beneath the Réunion region. In order to ensure the correctness of Mj-Pv phase transformation, we thoroughly investigated the Ps conversions. Results indicate a complex or broader phase conversion at the 660 km discontinuity where we observed the dominance of non-olivine phase transformations^45,48–50^. Moreover, this phase transformation requires extremely high temperatures. In order to ascertain this, we tried to estimate the temperatures from the observed MTZ topography variations.

Changes in the topography of MTZ discontinuities are directly or indirectly affected by temperature, phase transformation of minerals, composition, and convection^15,43,51–55^, among which temperature is one of the most important factors. The observed variations in the topography of MTZ discontinuities sampling the Réunion and the surrounding regions could be due to a thermal anomaly. The importance of using the 3D tomography models over 1D ones is that they account for velocity anomalies, if any, in the Fresnel zone of the ray paths. In addition, we calculated the excess temperatures based on the change in the depths to the 410 km and 660 km discontinuities. In order to calculate the excess temperatures, we considered two Clapeyron slopes (i) + 2.9 MPa/K^56^, (ii) + 4.0 MPa/K^57^ in the case of 410 km discontinuity and (i) − 2.6 MPa/K^58^, (ii) − 3.0 MPa/K^41^ in the case of 660 km discontinuity and the 1D thermochemical mantle model of Cobden et al.^59^. However, especially beneath the Réunion region, we majorly observed a depression in the 660 km discontinuity in a hot environment suggesting the dominance of Mj-Pv phase transformation. Hence, we considered a positive Clapeyron slope of + 1.3 MPa/K^17^ in the case of 660 km discontinuity beneath the Réunion region. Though the Clapeyron slope for the Mj-Pv phase at the 660 km discontinuity is not well constrained, a slope of + 1.3 MPa/K^17^ is considered to be reasonable. The Mj-Pv phase transformation is more temperature dependent since there is a wider pressure range at the depth of the 660 km discontinuity, between Majorite-garnet and Al-bearing Mg-rich perovskite. In addition, the Mj-Pv transformation takes place in a higher pressure range than the post-spinel phase boundary above 2073 K^17^. Also, it is inferred that bulk pyrolite with varying volume fractions of Mg-Pv (0–78%) may have different Clapeyron slopes in the temperature range of 1973–2073 K. Later, the magnitude of deviations in the 410 km and 660 km discontinuities are calculated by subtracting the global average depths of 410 km and 660 km from the observed depths to the MTZ discontinuities, to measure the corresponding excess temperatures. Positive deviations indicate a depression and negative deviations indicate an elevation in the corresponding MTZ discontinuities. The observed values along the profile indicate that deviations in the 410 km are from -40 km to +35 km and those in the 660 km discontinuity are from -37 km to +31 km. Moreover, especially beneath the Réunion hotspot region, the amount of deviation in 410 km varies from +6 km to +35 km and that in the 660 km varies from -15 km to +31 km. Further, we calculated the averaged deviations in the 410 km and 660 km discontinuities to calculate the excess temperatures at 410 km and 660 km discontinuities as per the chosen Clapeyron slopes, which are listed in table S3. The measured values indicate that there is an excess temperature of $\sim$193.8 K in the case of + 2.9 MPa/K Clapeyron slope and $\sim$140.8 K in the case of + 4.0 MPa/K slope at the 410 km discontinuity beneath the Réunion. The excess temperature estimated by considering a positive Clapeyron slope of + 1.3 MPa/K at 660 km discontinuity indicates an average of $\sim$215.4 K at this discontinuity. These excess temperatures agree with the calculated excess temperatures of plumes in the region of the Indian Ocean viz. 174 K in the hotspot region of Réunion, 232 K in the case of Crozet hotspot region^60^ and 235 K in the case of other hotspots in the Indian Ocean^61^. The excess temperatures, an average temperature of 215.4 K in the case of depressed 660 km discontinuity beneath the Réunion hotspot, support the Mj-Pv phase transformation. The Mj-Pv phase transformation occurs only when a minimum excess temperature is 200 to 300 K or above^53^. The calculated excess temperatures at the 660 km discontinuity beneath the Réunion corroborate with the results of Jenkins et al.^53^.

**Fig. 4:**
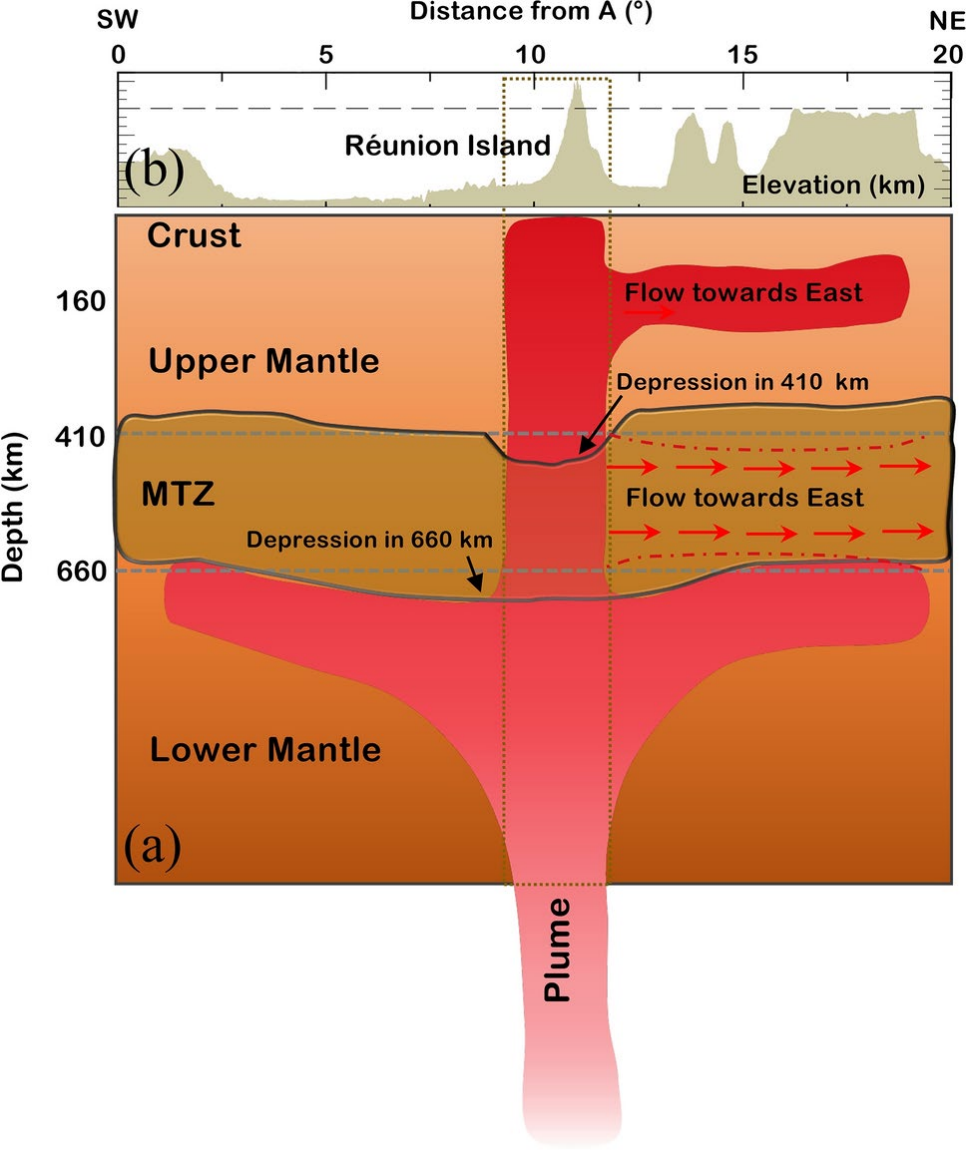
(**a**) Schematic architecture of the Réunion plume and the observed depths to the mantle transition zone (MTZ) discontinuities along profile AA* (SW-NE) (Reg-2 in Fig. 3). The dotted box indicates the Réunion Island region. The brown dotted lines indicate the observed depths to the mantle transition zone on the eastern side of Réunion, sampling the oceanic region (Reg-3 in Fig. 3). The flow of hot material towards the eastern side is indicated by red arrows. (**b**) The topography/bathymetry along profile AA*. The long dashed line indicates an elevation of 0 km.

Our results clearly indicate that the MTZ is affected due to the impingement of the rising Réunion plume. The depression of both discontinuities suggests Mj-Pv phase transformation at the 660 km discontinuity, which is possible in the presence of plumes, where the temperatures are sufficient for such a transformation. We postulate that the plume has initially hit the 660 km discontinuity in a broader region where we observe a depression in the 660 km discontinuity. This suggests that the rising Réunion plume is horizontally spread near the 660 km discontinuity, as shown in Fig.4. Further, it followed a columnar structure above this depth and hit the 410 km discontinuity, as revealed by a depression in the 410 km discontinuity exactly beneath the Réunion (Fig. 4). Also, results beneath the eastern side of Réunion Island (Reg-3 in Fig. 3) show a thin MTZ with a depressed 410 km and an elevated 660 km discontinuity, indicating a channelled flow of hot material from the hotspot towards the eastern side. The schematic architecture of the Réunion hotspot as per our results is shown in Fig. 4. The tomographic results are also in agreement with our results^62^. However, most of the global and regional tomographic models are unable to image the exact track of the plume from CMB to the surface. Moreover, the global and regional tomographic results indicate a deviation in the path of the plume towards the eastern side of the plume location above the MTZ. The latest study by Mazzullo et al.^8^ deciphered the mantle structure around the Réunion region by analysing Rayleigh waves recorded at 57 ocean bottom seismometers and 23 land stations. Their results clearly indicate the existence of a low shear wave velocity anomaly below the Rodrigues ridge and a low-velocity anomaly in the depth range of 150–300 km connected to the strong low-velocity anomaly beneath the Mascarene Basin^10^. Further, they interpreted that there could be a connection between the upwelling of Réunion and the central Indian ridge based on the east-west oriented azimuthal anisotropy. Also, the results of Rayleigh-wave tomography^20^ and shear wave splitting of SKS phases^9^ confirm the presence of eastward channelized flow from the hotspot to the central Indian ridge. These results suggest lateral transport of material from the Réunion plume to the CIR via Rodrigues, through the asthenospheric channel flow at a depth of 150-300 km. Results from the present study combined with the existing ones prompt us to suggest a schematic model for the architecture of the Réunion hotspot/plume, as in Fig. 4.

## Conclusions

3D-depth migration of good quality PRFs computed using data from 40 Ocean bottom seismometers and 43 land stations was performed to image the topography of the MTZ in and around the Réunion hotspot. Results reveal a thin MTZ owing to a depression of the 410 km discontinuity and elevation of the 660 km discontinuity beneath Madagascar, its surroundings, the eastern and south-eastern sides of Réunion, sampling the oceanic region. This provides clear evidence of a hot anomaly in the MTZ depth range. However, results beneath the Réunion indicate a depressed 410 km discontinuity exactly beneath the Réunion hotspot and a depressed 660 km discontinuity beneath a broader region i.e., underneath and surrounding regions of the Réunion hotspot along profile AA*. These results indicate existence of hot anomalies in the MTZ sourced from the plume and reveal evidence for Majorite-garnet (Mj) to Perovskite (Pv) phase transformation at the 660 km discontinuity. We propose that a rising Réunion plume initially impinged the 660 km discontinuity and is spread horizontally. Further, it followed a columnar structure above 660 km depth and interacted with the 410 km discontinuity.

## Supplementary Information

Supplementary Information.

## Data Availability

The waveform data can be downloaded from the Incorporated Research Institutions for Seismology (IRIS) data centre (https://ds.iris.edu/gmap/).
