# Supplementary material for: Inference of a plume conduit beneath the Réunion Island from 3D migration of Ps conversions from the mantle transition zone
Source: Sci Rep. 2025 Mar 29;15:10884. doi: 10.1038/s41598-025-94831-3 (PMC11954861; doi:10.1038/s41598-025-94831-3)
Supplement: Supplementary file 1 — Supplementary Information. [file 41598_2025_94831_MOESM1_ESM.pdf]

Supplementary Material for
Inference of a plume conduit beneath the Réunion Island from 3D Migration of Ps conversions from the Mantle Transition Zone

B. Padma Rao[1] and M. Ravi Kumar[2]
[1]MoES-National Centre for Earth Science Studies, Govt. of India, Thiruvananthapuram, India.
[2]CSIR-National Geophysical Research Institute, Hyderabad, India.

Contents of this file

1. Figures S1 to S8
2. Tables S1 to S3

Introduction

The supporting material contains eight figures and three tables, which are used in the 'Data and Methods' and 'Results and Discussion' sections.

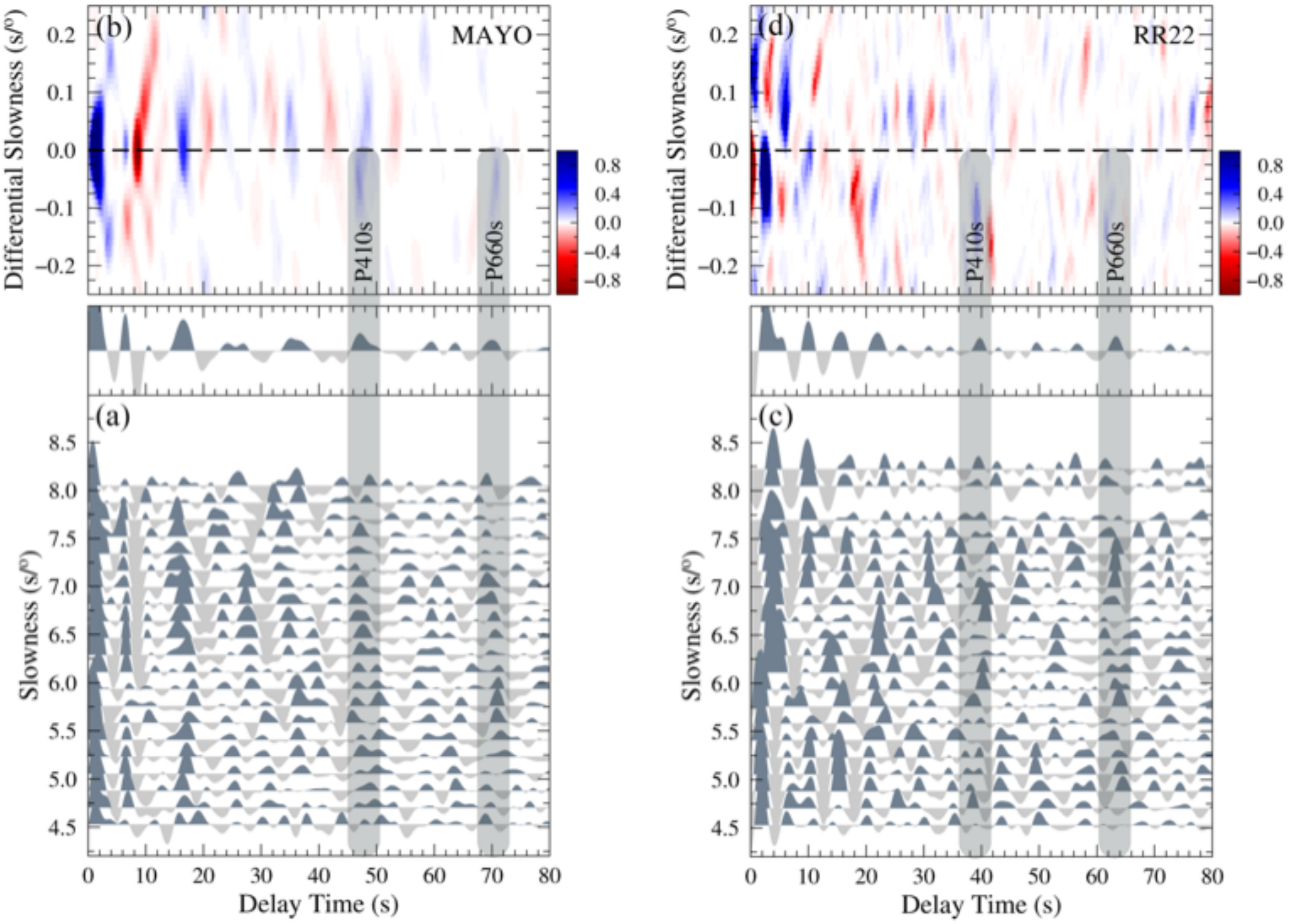

Figure S1: (a, c) Examples of Q(SV) component P-receiver functions (PRFs) stacked in narrow slowness bins, along with the final summation trace and (b, d) the corresponding differential slowness stacks at stations MAYO and RR22. The color palette table represents the 4th root of the stacked amplitudes of the PRFs, which are linear moveout corrected for differential slowness. The shaded regions in each panel indicate the observed delay times of the 410 km and 660 km discontinuities.

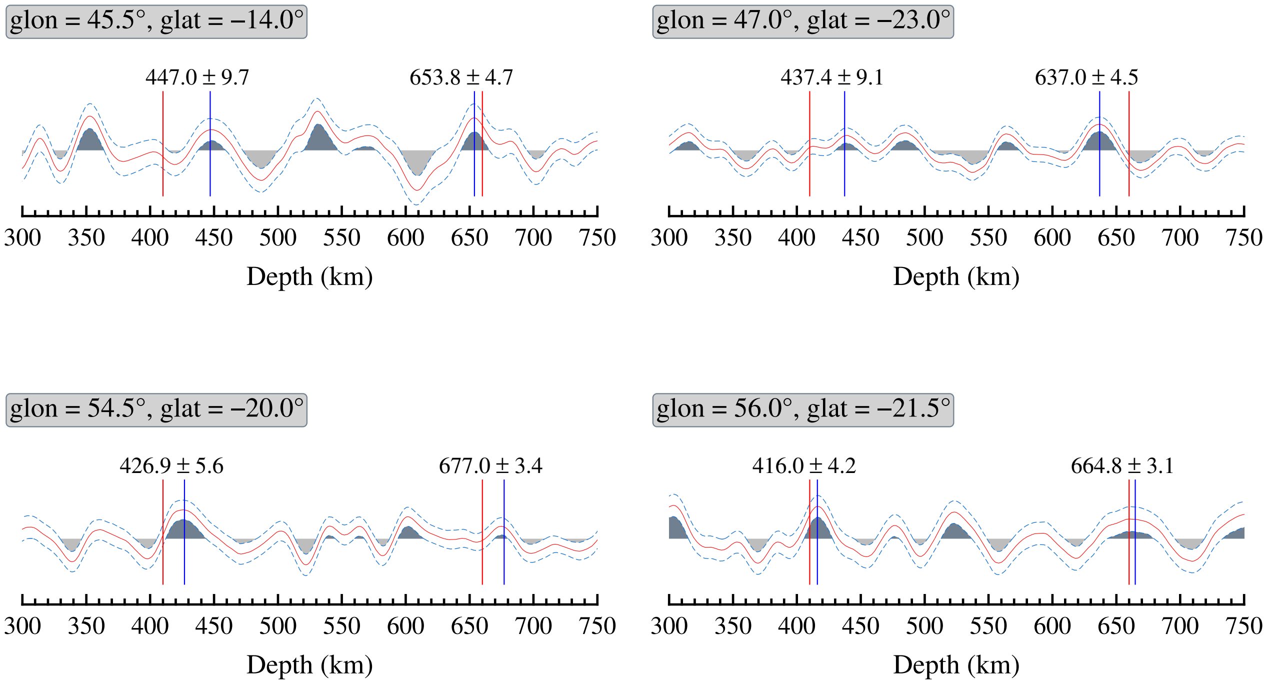

Figure S2. Examples of stacked, depth transformed PRFs migrated using the GyPSuM 3D velocity model at the corresponding grids whose longitude and latitude are specified. The red and blue vertical lines indicate the theoretical and observed depths to the 410 km and 660 km discontinuities and light blue dotted lines around the stacks (red) show one sigma error bounds.

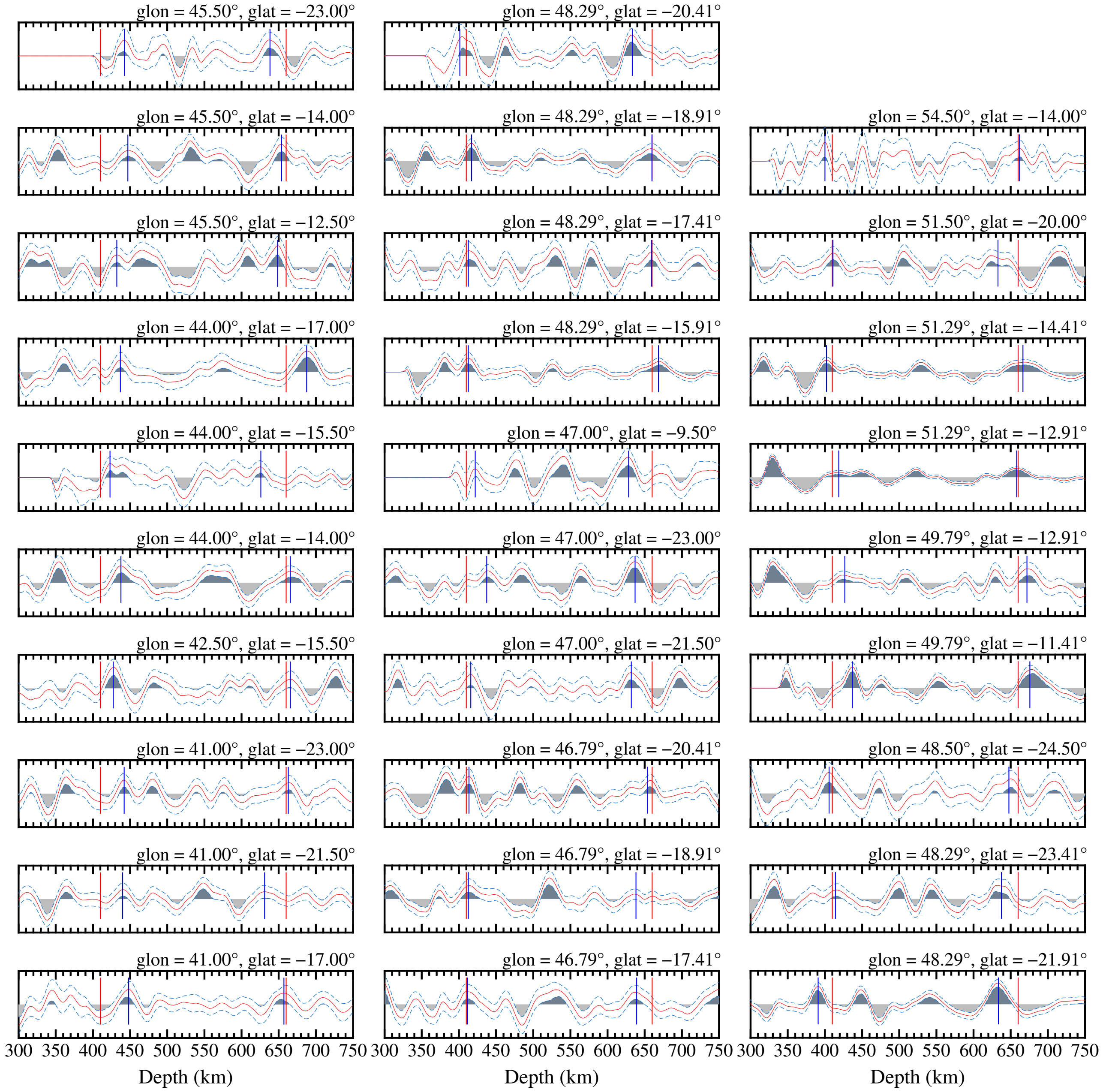

Figure S3: Stacked depth transformed PRFs migrated using the GyPSuM 3D velocity model at the corresponding grids whose longitude and latitude are specified, sampling Madagascar and its surrounding region. Red and blue vertical lines indicate the theoretical and observed depths to the 410 km and 660 km discontinuities and light blue dotted lines around the stacks (red) show one sigma error bounds.

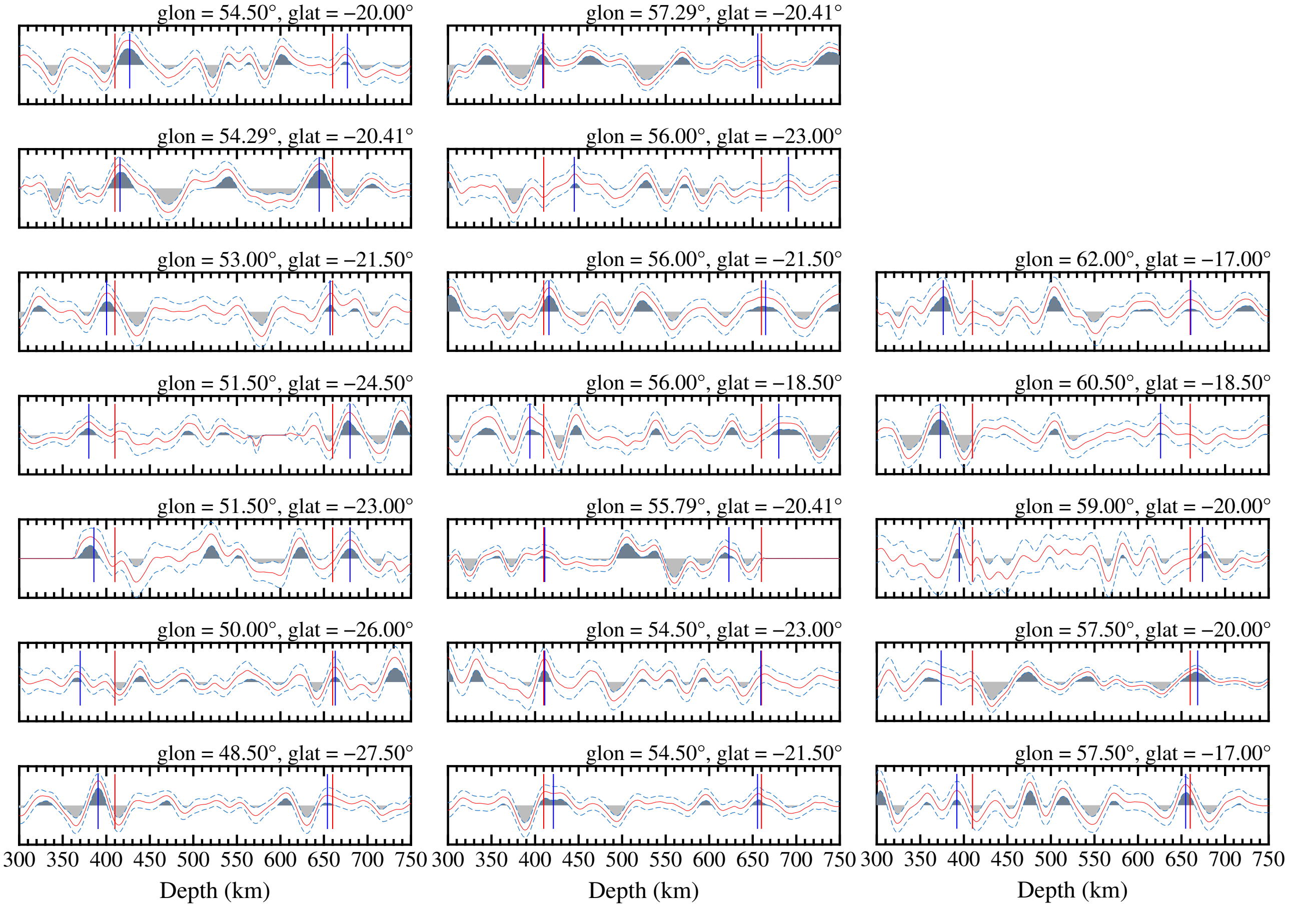

Figure S4: Stacked depth transformed PRFs migrated using the GyPSuM 3D velocity model, at the corresponding grids whose longitude and latitude are specified, sampling the region beneath and around the Réunion and Mauritius Islands along profile AA*. Red and blue vertical lines indicate the theoretical and observed depths to the 410 km and 660 km discontinuities and light blue dotted lines around the stacks (red) show one sigma error bounds.

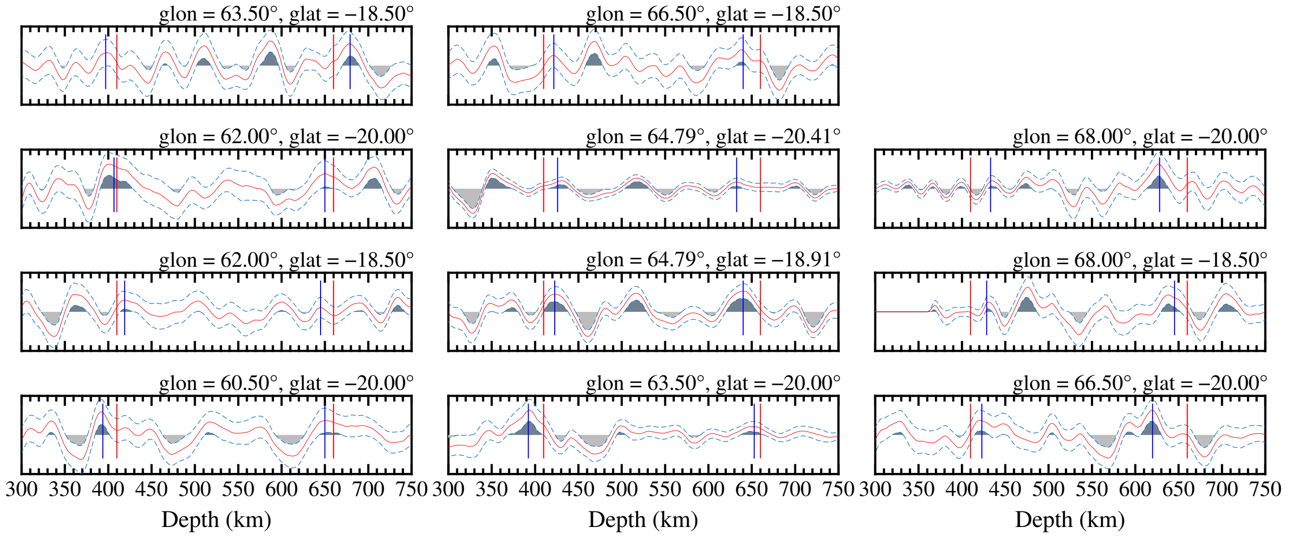

Figure S5: Stacked depth transformed PRFs migrated using the GyPSuM 3D velocity model at the corresponding grids whose longitude and latitude are specified, sampling the eastern side of the Réunion and Mauritius Islands. Red and blue vertical lines indicate the theoretical and observed depths to the 410 km and 660 km discontinuities and light blue dotted lines around the stacks (red) show one sigma error bounds.

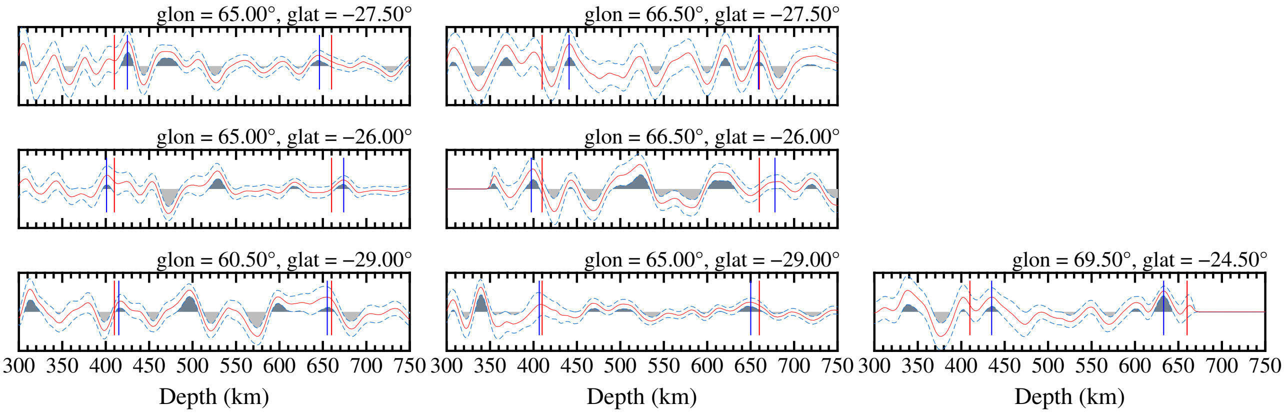

Figure S6: Stacked depth transformed PRFs migrated using the GyPSuM 3D velocity model at the corresponding grids whose longitude and latitude are specified, sampling the south-eastern side of the Réunion and Mauritius Islands. Red and blue vertical lines indicate the theoretical and observed depths to the 410 km and 660 km discontinuities and light blue dotted lines around the stacks (red) show one sigma error bounds.

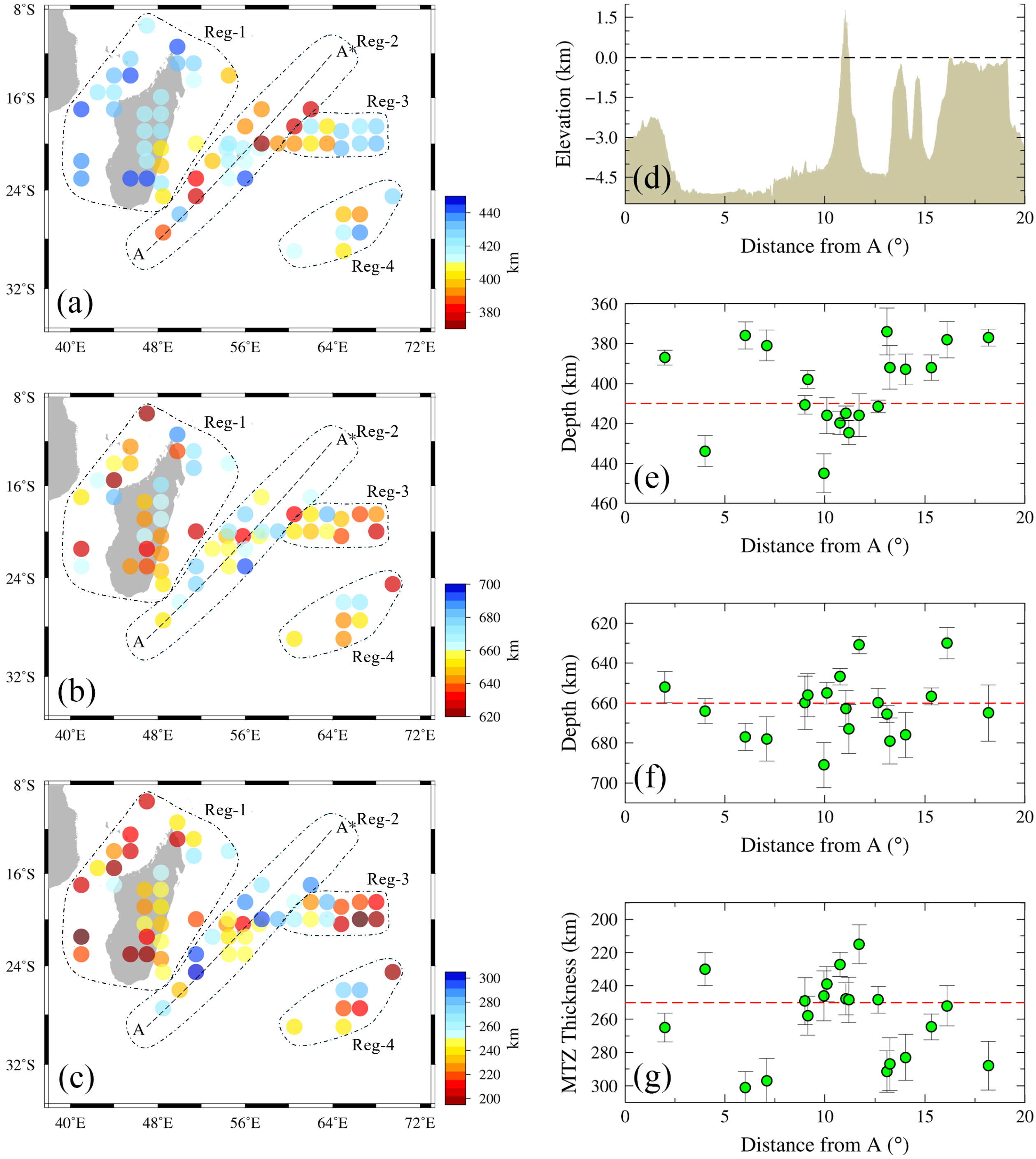

Figure S7: Variations in the depths to the (a) 410km, (b) 660km discontinuities and (c) mantle transition zone thickness, determined from depth migrated PRFs using the LLNL-G3D-JPS 3D velocity model. (d) The topography/bathymetry variations along profile AA*. Variations in the depths to the (e) 410km discontinuity, (f) 660km discontinuity and (g) mantle transition zone thickness along profile AA*. The dashed horizontal red lines indicate the (e) 410km depth, (f) 660km depth and (g) global average of the mantle transition zone thickness. Reg-1: The region beneath Madagascar and its surroundings; Reg-2: The region beneath the Réunion, Mauritius Islands and their surroundings along profile AA*; Reg-3: The eastern side of the Réunion and Mauritius Islands, sampling the oceanic region and Reg-4: Southeastern side of the Réunion, sampling the oceanic region.

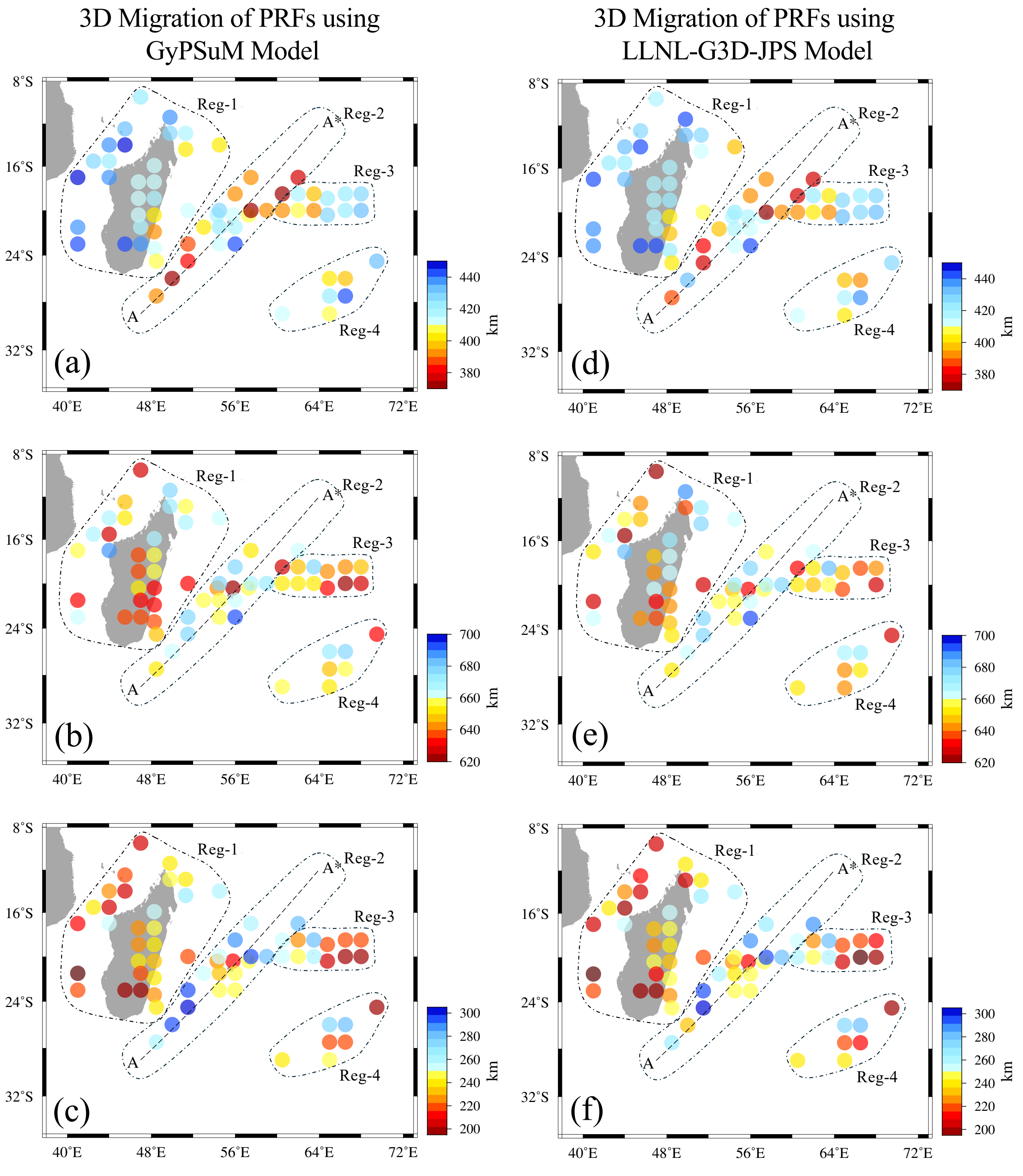

Figure S8: The variations in the depths to the (a) 410km, (b) 660km discontinuities and (c) mantle transition zone thickness, determined from PRFs depth migrated using the GyPSuM 3D tomography velocity model. The variations in the depths to the (d) 410km, (e) 660km discontinuities and (f) mantle transition zone thickness, determined from PRFs depth migrated using the LLNL-G3D-JPS 3D tomography velocity model. Reg-1: The region beneath Madagascar and its surroundings; Reg-2: The region beneath the Réunion, Mauritius Islands and their surroundings along profile AA*; Reg-3: The eastern side of the Réunion and Mauritius Islands, sampling the oceanic region and Reg-4: Southeastern side of the Réunion, sampling the oceanic region.

Table S1: Mean depths to the 410 km, 660 km discontinuities after 3D velocity correction using the GyPSuM tomography model and the mantle transition zone (MTZ) thickness along with the corresponding standard deviations.

S. No.	Grid Longitude (°)	Grid Latitude (°)	Mean d410 Depth (km)	σ_{d410} (km)	Mean d660 Depth (km)	σ_{d660} (km)	MTZ Thickness (km)	σ_{MTZ} (km)	No. of PRFs
**Reg-1: The region beneath Madagascar and its surroundings**									
1	46.79	-17.41	411.6	11.2	639.1	7.0	227.5	13.2	66
2	46.79	-18.91	412.6	14.1	638.2	7.44	225.6	9.32	32
3	46.79	-20.41	413.5	6.08	653.9	9.1	240.4	7.08	144
4	48.29	-15.91	412.6	6.52	668.5	4.2	255.9	6.72	42
5	48.29	-17.41	412.7	6.3	658.9	7.2	246.2	9.6	75
6	48.29	-18.91	417.0	2.8	659.7	5.5	242.7	6.2	148
7	48.29	-20.41	401.3	6.3	633.3	3.6	232.0	7.3	126
8	48.29	-21.91	390.9	6.44	633.4	3.1	242.5	6.56	357
9	48.29	-23.41	414.1	6.8	637.5	6.4	223.4	7.28	49
10	49.79	-11.41	436.9	2.5	675.8	3.7	238.9	4.5	91
11	49.79	-12.91	426.7	7.2	671.8	8.44	245.1	8.92	100
12	51.29	-12.91	418.6	14.1	658.1	4.4	239.5	14.8	481
13	51.29	-14.41	402.2	3.2	666.4	8.4	264.2	9.0	271
14	41.00	-17.00	448.0	14.32	657.0	7.5	209.0	14.64	24
15	41.00	-21.50	440.0	10.52	631.0	10.52	191.0	14.88	112
16	41.00	-23.00	442.0	11.84	662.8	8.0	220.8	12.24	113
17	42.50	-15.50	427.3	7.5	665.7	6.4	238.4	9.9	80
18	44.00	-14.00	437.7	4.1	665.5	6.0	227.8	7.2	51
19	44.00	-15.50	423.0	12.0	626.0	8.56	203.0	9.84	67
20	44.00	-17.00	437.0	6.56	687.6	4.5	250.6	6.8	194
21	45.50	-12.50	432.1	10.7	648.4	4.0	216.3	11.4	214
22	45.50	-14.00	447.0	9.7	653.8	4.7	206.8	11.7	36
23	45.50	-23.00	442.5	3.3	638.2	9.2	195.7	9.7	21
24	47.00	-21.50	416.0	7.5	632.0	12.44	216.0	12.8	104
25	47.00	-23.00	437.4	9.1	637.0	4.5	199.6	10.2	248
26	47.00	-9.50	422.0	10.6	628.4	8.4	206.4	13.5	30
27	48.50	-24.50	405.6	4.5	647.5	14.3	241.9	15.0	463
28	51.50	-20.00	410.9	4.5	632.8	11.3	221.9	12.2	54
29	54.50	-14.00	400.0	6.44	662.0	14.7	262.0	8.68	24
**Reg-2: The region beneath the Réunion, Mauritius Islands and their surroundings along profile AA***									
1	48.50	-27.50	390.6	3.3	654.0	8.5	263.4	9.1	30
2	50.00	-26.00	370.0	7.52	663.0	6.44	293.0	9.92	20

3	51.50	-24.50	380.0	6.28	680.0	7.36	300.0	9.68	15
4	51.50	-23.00	385.8	7.1	680.0	11.52	294.2	11.88	25
5	54.50	-23.00	411.2	3.2	659.2	6.16	248.0	6.32	398
6	53.00	-21.50	400.3	3.8	657.0	13.2	256.7	13.7	118
7	56.00	-23.00	445.0	11.3	691.0	13.1	246.0	6.88	214
8	54.50	-21.50	421.0	12.6	655.5	6.9	234.5	14.4	588
9	54.29	-20.41	415.8	5.7	644.5	4.0	228.8	7.0	28
10	56.00	-21.50	416.0	4.2	664.8	3.1	248.8	11.9	2230
11	54.50	-20.00	426.9	5.6	677.0	3.4	250.1	9.64	452
12	55.79	-20.41	411.2	10.4	622.6	10.5	211.4	14.8	156
13	57.29	-20.41	408.9	2.9	655.8	7.36	246.9	7.44	464
14	57.50	-20.00	374.0	6.4	668.5	4.4	294.5	7.7	809
15	56.00	-18.50	394.0	10.28	680.0	7.92	286.0	13	195
16	59.00	-20.00	394.8	6.9	674.1	7.9	279.3	10.5	48
17	57.50	-17.00	392.0	6.04	654.7	4.8	262.7	6.32	69
18	60.50	-18.50	373.0	10.6	626.0	12.4	253.0	6.52	45
19	62.00	-17.00	376.3	4.0	660.6	7.4	284.3	8.4	14
**Reg-3: The eastern side of the Réunion and Mauritius Islands, sampling the oceanic region**									
1	64.79	-18.91	422.7	6.56	640.0	4.9	217.3	6.84	121
2	64.79	-20.41	426.0	6.08	632.5	8.76	206.5	10.68	76
3	60.50	-20.00	393.7	8.0	650.0	14.3	256.3	6.56	92
4	62.00	-18.50	419.0	10.44	645.0	8.48	226.0	13.44	94
5	62.00	-20.00	406.6	9.3	650.0	6.84	243.4	7.8	119
6	63.50	-18.50	397.0	13.4	679.0	10.0	282.0	6.72	62
7	63.50	-20.00	392.2	6.8	652.8	7.5	260.6	10.1	61
8	66.50	-18.50	421.6	7.4	640.0	9.4	218.4	11.9	193
9	66.50	-20.00	423.0	12.8	620.0	3.9	197.0	13.4	154
10	68.00	-18.50	428.6	12.6	645.3	7.36	216.7	8.92	46
11	68.00	-20.00	433.0	14.7	628.0	7.7	195.0	6.64	30
**Reg-4: The south-eastern side of the Réunion, sampling the oceanic region**									
1	60.50	-29.00	415.0	8.08	655.0	9.0	240.0	8.84	37
2	65.00	-26.00	401.0	10.1	674.0	6.44	273.0	7.6	22
3	65.00	-27.50	425.0	10.3	646.0	6.24	221.0	7.48	128
4	65.00	-29.00	406.6	6.5	650.1	5.5	243.5	8.5	86
5	66.50	-26.00	397.5	6.2	678.0	6.52	280.5	6.96	37
6	66.50	-27.50	441.0	11.32	659.0	6.56	218.0	13.08	126
7	69.50	-24.50	435.0	6.44	633.0	13.7	198.0	8.44	27

Table S2: Mean depths to the 410 km, 660 km discontinuities after 3D velocity correction using the LLNL-G3D-JPS tomography model and the mantle transition zone (MTZ) thickness along with the corresponding standard deviations.

S. No.	Grid Longitude (°)	Grid Latitude (°)	Mean d410 Depth (km)	σ_{d410} (km)	Mean d660 Depth (km)	σ_{d660} (km)	MTZ Thickness (km)	σ_{MTZ} (km)	No. of PRFs
**Reg-1: The region beneath Madagascar and its surroundings**									
1	46.79	-17.41	417.6	10.7	648.8	11.2	231.2	6.2	66
2	46.79	-18.91	416.3	14.4	641.1	7.24	224.8	9.24	32
3	46.79	-20.41	418.1	6.36	663.2	8.6	245.1	7.24	144
4	48.29	-15.91	419.6	7.08	674.4	4.5	254.8	7.32	42
5	48.29	-17.41	417.9	6.4	664.0	5.8	246.1	8.6	75
6	48.29	-18.91	421.1	2.9	663.5	5.5	242.3	6.2	148
7	48.29	-20.41	404.6	7.0	640.5	3.7	235.9	7.9	126
8	48.29	-21.91	396.7	2.6	640.5	3.4	243.8	4.3	356
9	48.29	-23.41	420.7	7.4	647.5	8.8	226.8	8.2	49
10	49.79	-11.41	444.6	2.2	686.8	3.7	242.1	4.3	91
11	49.79	-12.91	433.0	5.1	638.2	8.6	205.2	8.84	100
12	51.29	-12.91	428.1	6.7	670.4	4.7	242.3	8.2	481
13	51.29	-14.41	411.8	3.7	679.4	10.3	267.6	10.9	271
14	41.00	-17.00	444.0	10.56	652.0	8.3	208.0	11.08	24
15	41.00	-21.50	439.0	7.92	629.0	10.84	190.0	13.44	112
16	41.00	-23.00	440.0	9.36	661.4	8.3	221.4	9.96	113
17	42.50	-15.50	423.0	11.6	660.1	5.6	237.1	12.9	80
18	44.00	-14.00	434.3	4.7	658.8	4.9	224.5	6.8	52
19	44.00	-15.50	422.0	6.84	621.0	10	199.0	12.16	67
20	44.00	-17.00	432.0	11.4	682.7	5.2	250.7	12.5	194
21	45.50	-12.50	427.7	10.6	642.7	5.4	215.0	11.9	215
22	45.50	-14.00	444.0	12.5	648.2	4.8	204.2	13.4	38
23	45.50	-23.00	443.4	4.2	640.4	9.1	197.0	10.0	21
24	47.00	-21.50	418.9	5.7	634.0	11.92	215.1	12.16	106
25	47.00	-23.00	442.0	14.3	638.2	4.5	196.2	14.9	248
26	47.00	-9.50	418.0	10.8	624.9	6.5	206.9	12.6	30
27	48.50	-24.50	404.0	6.9	652.0	10.7	248.0	12.7	463
28	51.50	-20.00	408.0	5.3	628.0	6.6	220.0	6.92	54
29	54.50	-14.00	399.0	14.6	662.0	14.5	263.0	8.24	24
**Reg-2: The region beneath the Réunion, Mauritius Islands and their surroundings along profile AA***									
1	48.50	-27.50	387.0	3.7	652.0	7.8	265.0	8.6	30
2	50.00	-26.00	433.9	7.7	664.0	6.24	230.1	6.96	21
3	51.50	-24.50	376.0	6.84	677.0	6.72	301.0	9.6	15
4	51.50	-23.00	381.0	7.7	678.0	11.08	297.0	11.52	25
5	54.50	-23.00	410.7	4.6	659.8	13.3	249.1	14.1	457
6	53.00	-21.50	398.0	4.4	656.0	10.8	258.0	11.7	118

7	56.00	-23.00	445.0	9.8	691.0	11.3	246.0	15.0	224
8	54.50	-21.50	416.0	9.0	655.0	5.4	239.0	10.4	678
9	54.29	-20.41	419.7	5.9	646.8	4.1	227.1	7.2	28
10	56.00	-21.50	414.9	3.6	662.7	8.9	247.8	9.6	2348
11	54.50	-20.00	424.7	5.9	673.0	12.3	248.3	13.6	525
12	55.79	-20.41	415.9	10.7	630.9	4.4	215.0	11.6	156
13	57.29	-20.41	411.6	3.2	659.9	7.32	248.3	7.44	464
14	57.50	-20.00	374.0	11.7	665.5	4.2	291.5	12.5	991
15	56.00	-18.50	392.0	10.88	679.0	11.6	287.0	15.92	213
16	59.00	-20.00	393.0	7.8	676.0	11.4	283.0	13.9	49
17	57.50	-17.00	392.0	6.4	656.6	4.2	264.6	6.6	69
18	60.50	-18.50	378.0	9.1	630.0	7.8	252.0	12.0	37
19	62.00	-17.00	377.0	4.2	665.0	14.0	288.0	14.6	14
**Reg-3: The eastern side of the Réunion and Mauritius Islands, sampling the oceanic region**									
1	64.79	-18.91	428.8	7.84	645.9	4.9	217.1	8.08	122
2	64.79	-20.41	434.9	10.4	637.8	6.48	202.9	12.24	76
3	60.50	-20.00	393.0	7.8	652.0	6.6	259.0	7.32	91
4	62.00	-18.50	424.0	8.56	650.0	8.08	226.0	11.76	94
5	62.00	-20.00	402.0	8.7	648.0	11.6	246.0	14.5	119
6	63.50	-18.50	401.0	14.1	684.0	14.5	283.0	8.08	61
7	63.50	-20.00	392.7	6.8	655.5	6.2	262.8	9.2	62
8	66.50	-18.50	422.0	11.9	638.4	5.0	216.4	12.9	194
9	66.50	-20.00	427.0	10.8	619.0	7.3	192.0	13.0	154
10	68.00	-18.50	430.0	9.0	641.0	10.84	211.0	11.4	46
11	68.00	-20.00	432.0	12.1	627.3	4.2	195.3	12.8	30
**Reg-4: The south-eastern side of the Réunion, sampling the oceanic region**									
1	60.50	-29.00	410.0	7.28	650.0	7.0	240.0	7.8	36
2	65.00	-26.00	396.0	12.4	669.0	9.1	273.0	6.16	21
3	65.00	-27.50	420.0	9.7	641.0	6.92	221.0	7.92	128
4	65.00	-29.00	403.0	8.6	641.0	4.9	238.0	9.9	86
5	66.50	-26.00	393.0	3.7	675.0	11.04	282.0	11.12	36
6	66.50	-27.50	436.0	8.48	650.1	6.7	214.1	8.88	127
7	69.50	-24.50	430.0	6.12	628.0	6.6	198.0	9	28

Table S3: Excess temperatures at the 410 km and 660 km discontinuities calculated using a thermochemical model by Cobden et al. (2008). Two Clapeyron slopes (1) 2.9 MPa/K (Bina & Helffrich, 1994), (2) 4.0 MPa/K (Katsura et al., 2004) in the case of 410 km discontinuity and three Clapeyron slopes (3) +1.3 MPaK−1 (Hirose, 2002), (4) −2.6 MPa/K (Akaogi et al., 2007), (5) −3.0 MPa/K (Ito & Takahashi, 1989) in the case of 660 km discontinuity, were considered for this estimation.

**Average discontinuity depth in km**	**Average excess temperature in K**	
Beneath the Réunion along AA* profile		
425	2.9 MPaK^{-1} (1)	4.0 MPaK^{-1} (2)
	193.8	140.8
Beneath the south-western and north-eastern sides of the Réunion along AA* profile		
390	2.9 MPaK^{-1} (1)	4.0 MPaK^{-1} (2)
	-258.5	-187.7
**Average discontinuity depth in km**	**Average excess temperature in K**	
Beneath the Réunion along AA* profile		
667	+1.3 MPaK^{-1} (3)	
	215.4	
Beneath the broader region of the Réunion along AA* profile		
665	+1.3 MPaK^{-1} (3)	
	153.9	
Beneath the south-western and north-eastern sides of the Réunion along AA* profile		
649	-2.6 MPaK^{-1} (4)	-3.0MPaK^{-1} (5)
	169.2	146.7
